# Onion membrane: an efficient adsorbent for decoloring of wastewater

**DOI:** 10.1186/s40201-015-0170-6

**Published:** 2015-03-13

**Authors:** Samaneh Saber-Samandari, Jalil Heydaripour

**Affiliations:** Department of Chemistry, Eastern Mediterranean University, TRNC via Mersin 10, Gazimagusa, Turkey

**Keywords:** Adsorption, Cationic dye, Methylene blue, Onion membrane, Water treatment

## Abstract

**Background:**

Recently, researchers have tried to design synthetic materials by replicating natural materials as an adsorbent for removing various types of environmental pollutants, which have reached to the risky levels in nature for many countries in the world. In this research, the potential of onion membrane obtained from intermediate of onion shells for adsorption of methylene blue (MB) as a model cationic dye was exhibited.

**Methods:**

Before and after adsorption, the membrane was characterized by Fourier transform infrared spectroscopy (FTIR) and optical and scanning electron microscopy in order to prove its dye adsorption capability. The various experimental conditions affecting dye adsorption were explored to achieve maximum adsorption capacity.

**Results:**

The dye adsorption capacity of the membrane was found to be 1.055 g.g^−1^ with 84.45% efficiency after one hour and 1.202 g.g^−1^ with 96.20% efficiency after eight hours in contact with the dye solution (0.3 g.L^−1^). Moreover, the kinetic, thermodynamic and adsorption isotherm models were employed to described the MB adsorption processes. The results show that the data for adsorption of MB onto the membrane fitted well with the Freundlich isotherm and pseudo-second-order kinetic models. In addition, the MB adsorption from room temperature to ~50°C is spontaneous and thermodynamically favorable.

**Conclusions:**

Evidently, the high efficiency and fast removal of methylene blue using onion membrane suggest the synthesis of polymer-based membranes with similar physical and chemical properties of onion membrane as a valuable and promising wastewater decoloring agents in water treatment.

## Background

Many industrial production activities (e.g. dye, cosmetic, plastics, food, textile, planting, and mining) result in water pollution since they produce pollutants such as water-coloring agents and toxic heavy metals that are extremely harmful to people and the environment even at low concentrations. The removal of these compounds from wastewaters before discharging them into the environment is of great importance, since many dyes and their degradation products are toxic and carcinogenic, posing a serious hazard to the environment. The conventional methods used to treat colored effluents include photocatalytic degradation, microbiological decomposition, electrochemical oxidation, membrane filtration, and adsorption techniques [1]. Among these, adsorption is the most widely used method because of its efficiency, low cost, easy operation, simple design, less energy intensiveness, and non-toxicity. Recently, numerous approaches to develop adsorbents that are more effective have been studied [2]. Among these, adsorbents containing natural and synthetic polymeric materials, industrial by-products, agricultural wastes and biomass were applied for removal of dyes from aqueous solution [3-6].

Dyes can be classified into cationic, anionic and nonionic dyes. Cationic dyes are basic dyes while the anionic dyes include direct, acid, and reactive dyes [7]. However, cationic dyes are widely used in acrylic, wool, nylon, and silk dyeing, they considered as toxic colorants and can cause harmful effects such as allergic dermatitis, skin irritation, mutations and cancer [8]. Cationic dyes carry a positive charge in their molecule, furthermore they are water soluble and yield colored cations in solution [9]. Methylene blue, rhodamine B, and brilliant green are representative examples of cationic dyes [10]. Methylene blue (MB) is an important basic dye and widely used in the textile industry. Acute exposure to MB may cause increased heart rate, shock, vomiting, cyanosis, jaundice, quadriplegia, heinz body formation, and tissue necrosis in humans [11].

The main aim of this study is to exhibit the ability of dried onion membrane for removal of MB from aqueous solution. However, many researchers have studied the dye and metal adsorption capacity of several biomasses such as rice, corn and coconut husks [12-14], papaya seeds [15], watermelon [3] and onion skin [4], but to the best of our knowledge, there is no study relating the adsorption properties of onion membrane obtained from intermediate of onion shells. In this study, the adsorption of MB was confirmed using FTIR and optical and electron microscopy. In addition, the effect of various factors such as contact time, initial dye concentration, pH, temperature and adsorbent dose on the adsorption rate was examined. Finally, the adsorption of MB was analyzed by employing the adsorption kinetic, isotherm models, and thermodynamics.

## Experimental procedures

### Material

The onions were purchased from local markets in Famagusta (North Cyprus). Hydrochloric acid 37% (Merck), sodium hydroxide (Mediko Kimya), potassium chloride (Mediko Kimya), potassium hydrogen phthalate (Merck), sodium hydrogen carbonate (Aldrich), and potassium hydrogen phosphate (Merck) were used to prepare the buffer solutions with different pH values. Finally, MB (Aldrich) with a molecular formula of C_16_H_18_ClN_3_S was used without further purification.

### Adsorbent preparation

The onions were peeled, chopped and then the membranes were removed from the leaves. 1 gram of onion membrane was obtained from approximately 250 g onion. The membranes (~4 g/kg of onion) were rinsed and washed with distilled water to remove impurities. Then, it was dried at 60°C in an oven for one day. After that, the dried adsorbent was kept in the desiccator to avoid moisture adsorption.

### Swelling measurements

The swelling property of the membranes were investigated by immersing 0.06 g of membrane (15 × 15 mm^2^) in 250 mL of distilled water at room temperature (±20°C) in atmospheric conditions until swelling equilibrium was reached. Following the removal from the water, they were blotted with filter paper and weighed. Then, the swelling capacity was calculated using the following equation [16,17]:

1 $$ \mathrm{Swelling}\ \left(\%\right)=\frac{{\mathrm{W}}_2-{\mathrm{W}}_1}{{\mathrm{W}}_1}\times 100 $$

where W_1_ (g) and W_2_ (g) are the weights of the dried and swollen membranes, respectively.

### Dye adsorption

The initial aqueous MB solutions were prepared by dissolving 0.075 g of MB in 250 mL of deionized water (0.3 g.L^−1^). Then, the 0.06 g of dried onion membranes (15 × 15 mm^2^) were immersed in a prepared MB solution and shaken at 300 rpm for 10 hours at room temperature. During this period, 2 mL of solution was taken for further analysis frequently. Finally, the collected solutions were filtered and the amount of non-adsorbed dye ions in the solutions was determined spectrophotometrically using a UV-visible spectrophotometer at a wavelength of 668 nm [6]. The MB adsorption amount, capacity, and efficiency were calculated by the following equations:

2 $$ \mathrm{adsorption}\ \mathrm{amount}\ \left(\mathrm{g}.{\mathrm{L}}^{-1}\right)={\mathrm{C}}_{\mathrm{i}}-{\mathrm{C}}_{\mathrm{e}} $$

3 $$ \mathrm{adsorption}\ \mathrm{capacity}\ \left(\mathrm{g}.{\mathrm{g}}^{-1}\right)={\mathrm{q}}_{\mathrm{t}}=\frac{{\mathrm{C}}_{\mathrm{i}}-{\mathrm{C}}_{\mathrm{e}}}{\mathrm{W}}\times \mathrm{V} $$

4 $$ \mathrm{adsorption}\ \mathrm{efficency}\ \left(\%\right)=\frac{{\mathrm{C}}_{\mathrm{i}}-{\mathrm{C}}_{\mathrm{e}}}{{\mathrm{C}}_{\mathrm{i}}}\times 100 $$

where W is the mass of adsorbent in g, V is the volume of MB solution in L, and C_i_ and C_e_ are the initial and equilibrium concentrations in g.L^−1^, respectively.

In addition, the effect of variable conditions such as time, pH, adsorbent amount, and initial adsorbate concentration on the MB adsorption behavior of onion membranes was examined. For each case, one parameter was changed and analyzed and the other factors were kept constant. For instance, the influence of pH on adsorption was calculated by immersing 0.06 g of onion membrane in 250 mL of MB buffer solutions (0.3 g.L^−1^ concentration) with different pH values (3–11) and then shaken at room temperature (20°C) for eight hours (480 min).

Furthermore, the pH at point of zero charge (pHzpc), which shows the point that the acidic or basic functional groups do no contribute to the pH of the solution, was determined using the standard technique [18]. For this purpose, 50 mL of 0.01 mol.L^−1^ NaCl solution was placed in a 250 ml flask. The pH of the solutions in each flask was adjusted from 3–11 by adding either sodium hydroxide or hydrochloric acid solutions (0.1 mol.L^−1^). Then, 0.06 g of onion membrane (15 × 15 mm^2^) was immersed in each solution at 20°C and was allowed to equilibrate in an isothermal shaker. After a contact time of 24 hours, the suspensions were filtered through filter paper and the final pH values of supernatant were measured again using a pH meter. Lastly, the final pH values were plotted against the initial pH values. The pH at which the curve crosses the line final pH = initial pH was taken as the pHzpc of the onion membrane [19].

### Methods of characterization

A UV/VIS spectrophotometer (Lambda 25 UV/VIS, Perkin-Elmer, Llantrisant, UK) was used to determine the MB adsorption amounts by the onion membranes. The pH values of the solutions, which were used to investigate the effect of pH on adsorption, were checked by a pH meter (WTW InoLab, accuracy ± 0.1). To prove the adsorption of MB by the onion membranes, the FTIR (Perkin-Elmer, Llantrisant, UK) spectra of membranes before and after adsorption were observed in the range of 500–4000 cm^−1^. Images of the membranes were also taken with an optical microscope (MT9000 Polarizing Microscope, Meiji Techno Co. Ltd., Japan with Invenio 3S, Delta Pix Camera) and a scanning electron microscope (AIS2100, Seron Technology, Korea) operated at an acceleration voltage of 15 kV to study the change in surface morphology of the membrane after dye adsorption.

## Results and discussion

### Swelling properties of onion membrane

**Figure 1:**
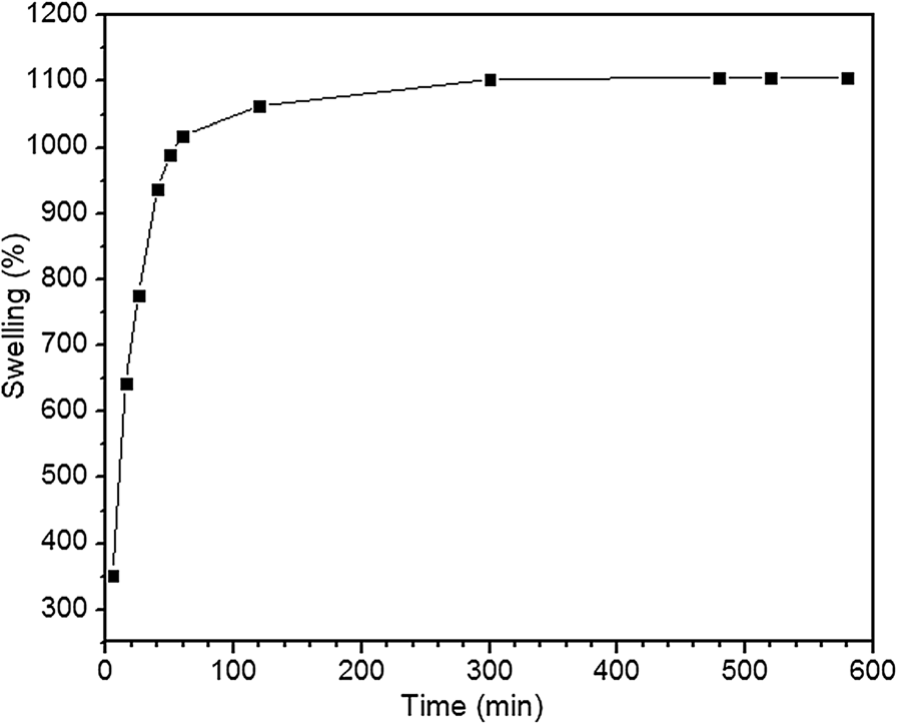
**Swelling behavior of the onion membrane was plotted as a function of time.** In this experiment, 0.06 g of membrane was immersed in 250 mL of water with a pH of 7.1 at 20°C.

The dynamic swelling behavior of the onion membranes over 10 hours (600 min) of immersion in water is shown in Figure 1. The swelling percentage increased up to 1,106% and then plateaued, with no big differences in water uptake with further increases in time. Initially, the water molecules were in contact with the membrane, then, they attacked and penetrated into the onion membrane cells. Obviously, this swelling system cannot continue forever, and by the increasing membrane-water interaction, the osmotic pressure difference might be reduced. Finally, the osmotic force at the equilibrium state was balanced with an elasticity force. It should be noted that the elasticity force prevents the deformation of the onion membrane cells by the stretching balance of the cells.

### Dye adsorption properties of onion membrane

**Figure 2:**
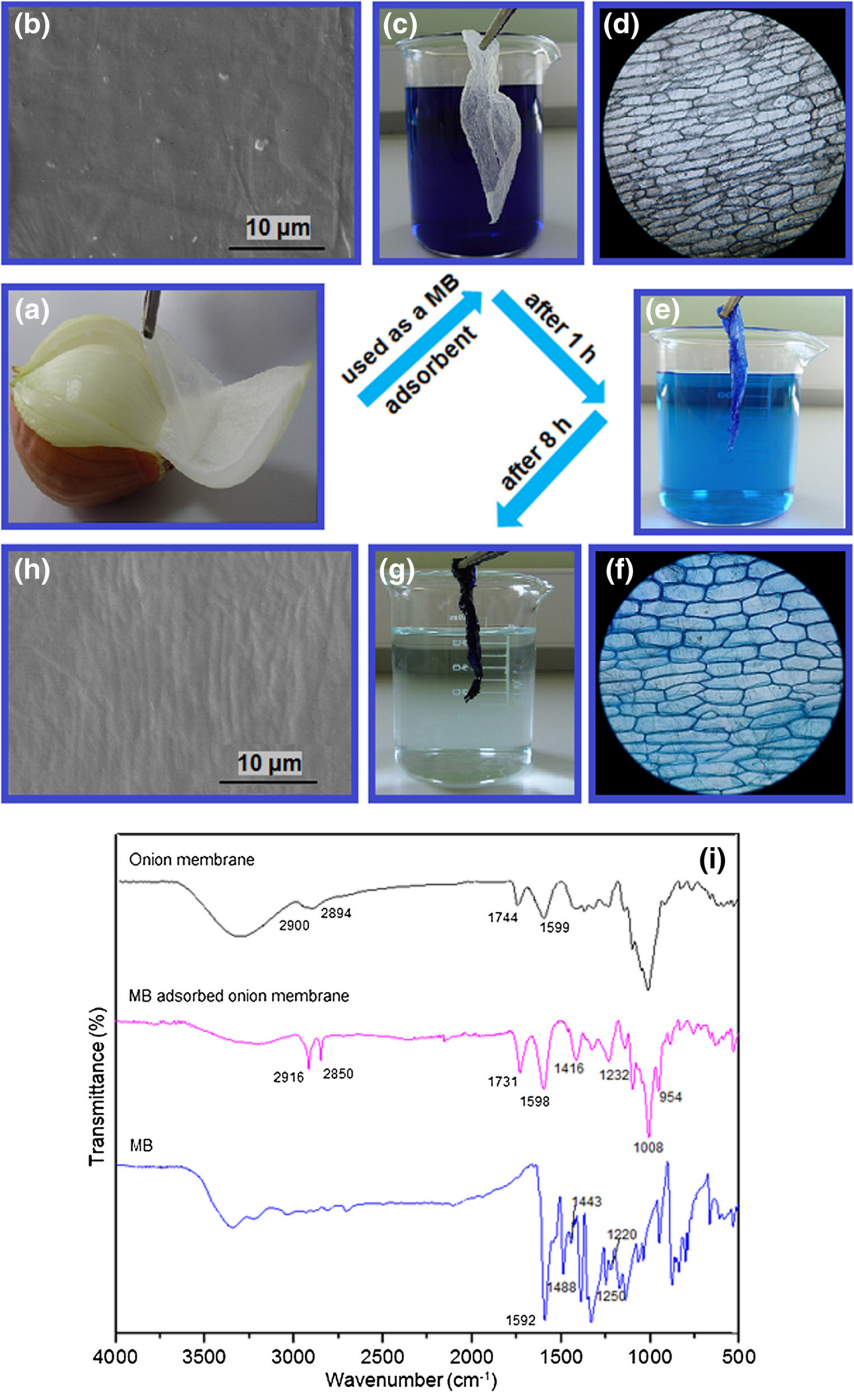
**(a-d) Digital photographs, (e and f) SEM images, (g and h) optical microscope images, and (i) FTIR spectra of the onion membrane before and after dye adsorption are shown.**

The MB adsorption capacity of the onion membranes was examined. Figure 2 shows the preparation of the membranes for MB adsorption (Figure 2a) and color changes in the membrane and dye solutions before and after adsorption during the first and eight hours of contact time (Figure 2b-d). In addition, the scanning electron microscopy (SEM) images of the membranes before and after adsorption of MB reflect their surface morphology (Figure 2e and f). As can be seen, before adsorption the membrane has a smooth surface, whereas it exhibits a coarse surface due to the presence of MB molecules after adsorption. In addition to this distinctive change in the surface of the membrane, the optical microscope images of the membrane before and after MB adsorption (Figure 2g and h) also revealed the adsorption of MB by the onion membrane.

The FTIR spectrum of the onion membrane after adsorption of the dye was compared with the spectra of pure MB and dried onion membrane before adsorption in Figure 2i. The several peaks in the spectrum of dye-adsorbed membrane related to the MB and membrane are merged and slightly shifted. The peaks at 2,894 cm^−1^, 2,900 cm^−1^, and 1,744 cm^−1^ due to the C-H stretching in an aromatic methoxyl group and C = O stretching of carbonyl group of onion membrane, respectively, became stronger and were shifted to 2,916 cm^−1^, 2,850 cm^−1^, and 1,731 cm^−1^ in the dye adsorbed membrane [20]. The sharp and strong peak at 1,592 cm^−1^ of MB due to the presence of C = C vibration and N-H bending was merged with a peak at 1,599 cm^−1^ of membrane and showed a broader peak at 1,598 cm^−1^ in the spectrum of the dye-adsorbed membrane [21]. Like the two peaks at 1,443 cm^−1^ and 1,488 cm^−1^, which indicates C = N stretching, the peaks at 1,220 cm^−1^ and 1,250 cm^−1^ of C = C stretching in aromatic rings of MB are merged and form broad peaks at 1,416 cm^−1^ and 1,232 cm^−1^, respectively, in the spectrum of the dye-adsorbed membrane [18]. Finally, the peaks corresponding to the C-O stretching of the onion membrane and the C-S bending of the MB rings appeared at 1,008 cm^−1^ and 954 cm^−1^, respectively [22]. The FTIR results accompanied with the supportive results of the SEM and the optical microscope confirmed the adsorption of MB by the onion membrane.

**Scheme 1:**
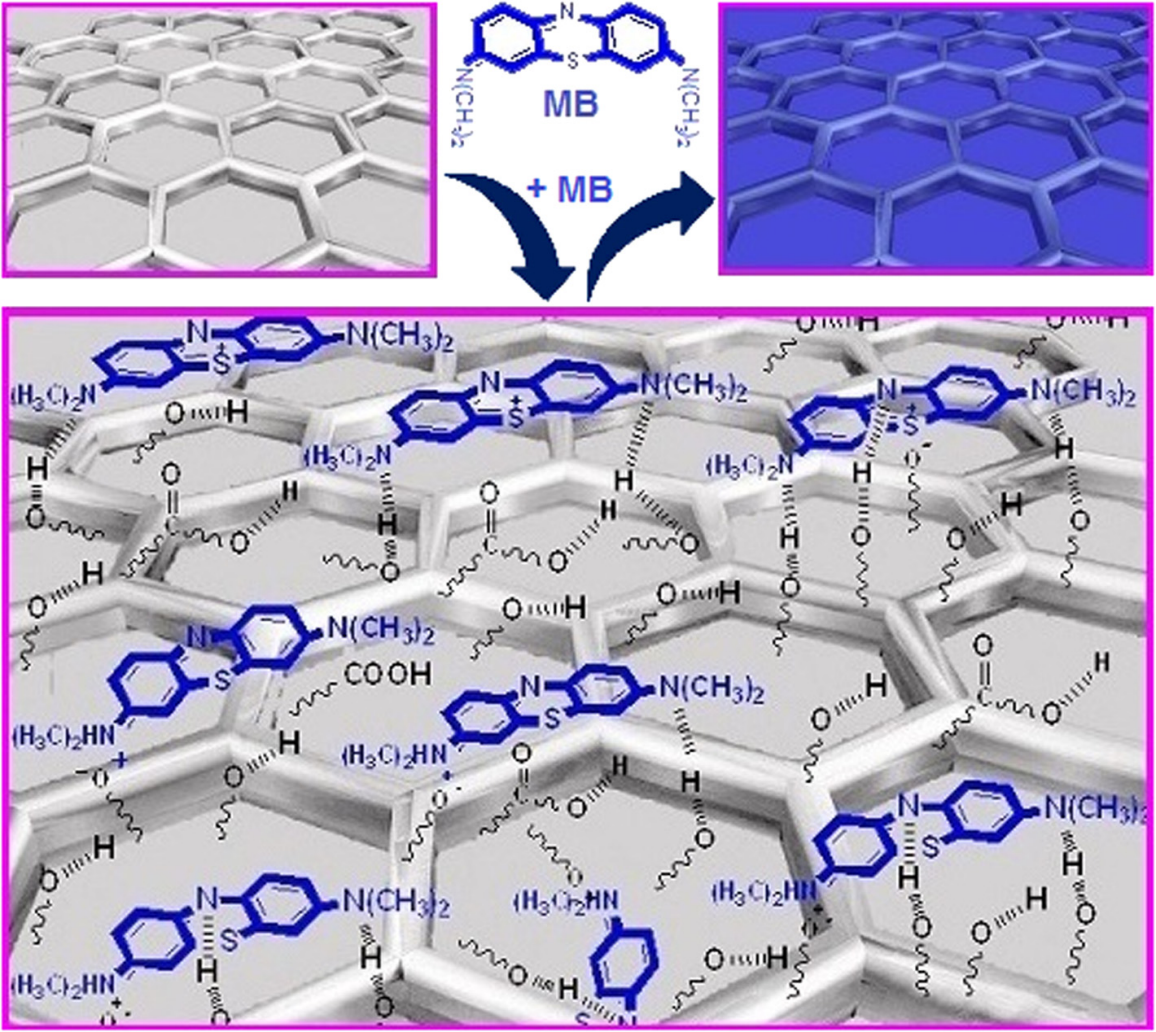
**Schematic illustration for the adsorption mechanism of MB by onion membrane.**

Onions contain protein (with –COOH and –NH_2_ groups), sugars, carbohydrate, and vitamins A, B_6_, and C (with –OH groups), minerals, and over 80% water [23]. As can be seen from Scheme 1, the onion membrane has several anionic groups such as –COOH and –OH. On the other hand, MB is a cationic dye consisting of = S– and –N(CH_3_)_2_ can become charged species and have ionic and dipole–dipole interactions with anionic groups in the surface of the onion membrane [24]. In addition, the = N– and –N(CH_3_)_2_ groups in the structure of MB can have hydrogen bonds with hydrogen atom of –COOH and –OH groups of the onion membrane. Therefore, the adsorbent can uptake MB very fast with high efficiency through the strong electrostatic attraction between the surface groups on the membrane and the cationic MB.

#### Effect of time on adsorption

**Figure 3:**
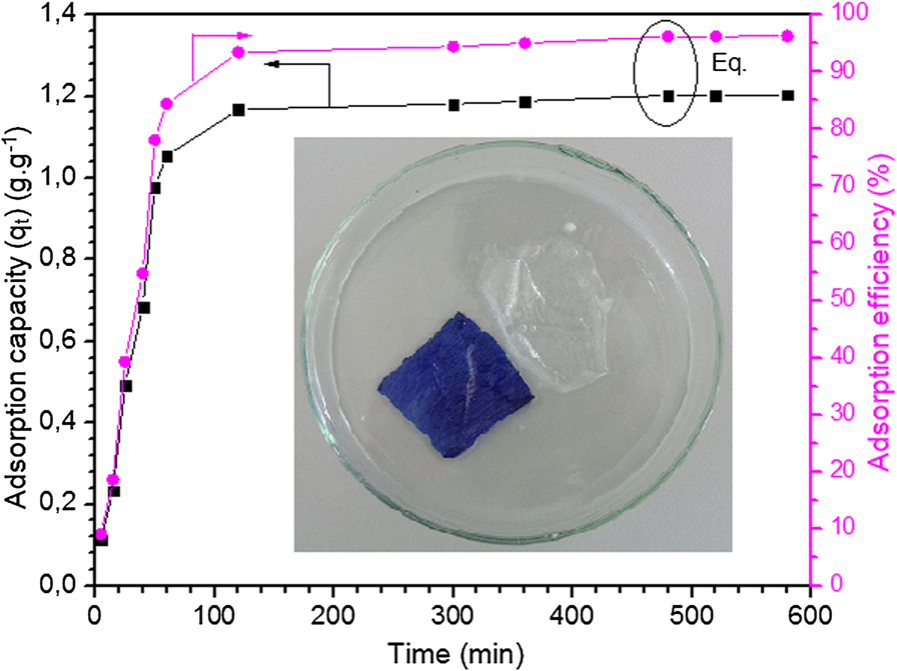
**Effect of time on MB adsorption capacity (g.g** ^**−1**^ **) and efficiency (%) of membrane was plotted.** In this experiment, dye molecules from a 0.3 g.L^−1^ dye solution (250 ml) with a pH of 7.1 at 20°C were taken up by 0.06 g of the membrane. Insert shows the onion membrane in swollen and dye-adsorbed state.

The adsorption performance of the onion membrane was evaluated by a batch equilibration technique as a function of time. As shown in Figure 3, the adsorption capacity of MB ions on the membrane increased rapidly during the first hour of contact and then became slower until equilibrium was reached after eight hours (480 min). The maximum adsorption was 1.055 g.g^−1^ with 84.45% efficiency after the first hour and 1.202 g.g^−1^ with 96.20% efficiency after eight hours. This behavior can be attributed to the larger surface area of the onion membrane at the initial stage of the adsorption process. Subsequently, as the surface sites became saturated, adsorption did not increase significantly with further contact time.

#### Adsorption kinetics

**Figure 4:**
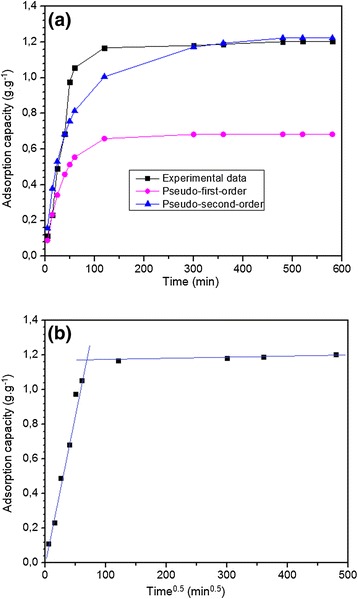
**Adsorption kinetics of MB on membrane by (a) pseudo-first-order and pseudo-second-order, and (b) intra-particle models kinetic was plotted.** In this experiment, dye molecules from a 0.3 g.L^−1^ dye solution (250 ml) with a pH of 7.1 at 20°C were taken up by 0.06 g of the membrane.

In order to investigate the possibility of using an adsorbent for a particular separation task and to determine the adsorption efficiency as well as the adsorption rate, the kinetic mechanism of the adsorption process was considered. The adsorption kinetics of the MB ions with the onion membranes was investigated using three kinetic models: pseudo-first-order, pseudo-second-order, and intra-particle diffusion, which are given in the following equations, respectively.

5 $$ \log \left({\mathrm{q}}_{\mathrm{e}}-{\mathrm{q}}_{\mathrm{t}}\right)= \log {\mathrm{q}}_{\mathrm{e}}-\frac{{\mathrm{k}}_1}{2.303}\mathrm{t} $$

6 $$ \frac{\mathrm{t}}{{\mathrm{q}}_{\mathrm{t}}}=\frac{1}{{\mathrm{k}}_2{\mathrm{q}}_{\mathrm{e}}^2}+\frac{\mathrm{t}}{{\mathrm{q}}_{\mathrm{e}}} $$

7 $$ {\mathrm{q}}_{\mathrm{t}}={\mathrm{k}}_3{\left(\mathrm{t}\right)}^{\frac{1}{2}}+{\mathrm{C}}_{\mathrm{i}} $$

where t is the time (min) and q_e_, q_t_, (g.g^−1^) and q_e_^2^ are the amounts of MB adsorbed by the onion membrane at equilibrium, at time t, and at maximum adsorption capacity, respectively. k_1_ (min^−1^), k_2_ (g.g^−1^.min^−1^), and k_3_ (g.g^−1^.min^−0.5^) are the adsorption rate constants of the pseudo-first-order, pseudo-second order and the intra-particle diffusion models, respectively. In addition, C_i_ (g.g^−1^) is the intra-particle diffusion constant, which is directly proportional to the boundary layer thickness. As shown in Table 1, the theoretical equilibrium adsorption capacity (1.3166 g.g^−1^) using the pseudo-second-order model compared well with the experimental data (1.2025 g.g^−1^), with a better R^2^ value. Moreover, Figure 4a shows the agreement between the experimental adsorption capacities with the calculated values of pseudo-second-order, which were obtained using the data in Table 1. Therefore, the agreement between experimental data and pseudo-second-order can prove the physical adsorption of MB on a highly heterogeneous onion membrane.

**Table 1:** **Comparison of kinetic models for adsorption of MB using onion membrane**

**Kinetic models and parameters**	**MB**
q_e_ exp. (g.g^−1^)	1.2025
**Pseudo-first order**	
K_1_ (min^−1^)	0.0278
q_e_ cal. (g.g^−1^)	0.6831
R^2^	0.8255
**Pseudo-second order**	
K_2_ × 10^−4^ (g.g^−1^.min^−1^)	0.0205
q_e_ cal. (g.g^−1^)	1.3166
R^2^	0.9893
**Intraparticle diffusion**	
K_3(1)_ (g.g^−1^.min^−1/2^)	0.1747
C_1_ (g.g^−1^)	0.3567
R^2^	0.9414
K_3(2)_ (g.g^−1^.min^−1/2^)	0.0086
C_2_ (g.g^−1^)	1.0266
R^2^	0.7264

According to Equation 7, if the intra-particle diffusion is the main rate-controlling step, the plot of q_t_ versus t^0.5^ should be linear and pass through the origin. However, the plot shown in Figure 4b did not pass through the origin and presented multilinearity, indicating the presence of two steps in the adsorption process. The intra-particle diffusion parameters for these steps are summarized in Table 1. The first linear segments can be attributed to the dye transfer from the solution onto the external surface or boundary layer of the onion membrane. The second step could be attributed to the final apparent equilibrium process, which reflects the intra-particle diffusion slowing down due to lowering the dye concentration in the solution [5]. As seen from the data in Table 1, the intra-particle diffusion constants of the two linear segments are not similar and the first step comprises the bigger k_3(1)_ value (0.1747 g.g^−1^.min^-0.5^) and the higher correlation coefficient 0.9414. This observation indicates that the adsorption of dye onto the onion membrane at the first section occurs more rapidly due to the availability of adsorption centers, then this is followed by the slow diffusion, which takes up to eight hours.

#### Effect of pH on adsorption

**Figure 5:**
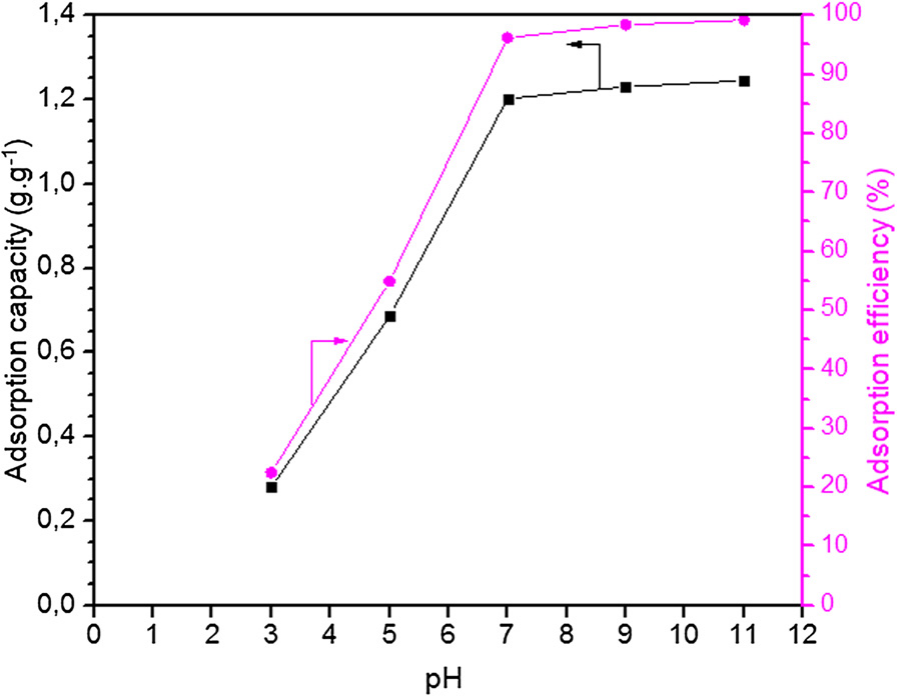
**Effect of pH on the MB adsorption capacity (g.g** ^**−1**^ **) and efficiency (%) of membrane was plotted.** In these experiments, 0.06 g of membrane adsorbed dye molecules from a 0.3 g.L^−1^ dye solution (250 ml) at 20°C for 8 hours.

The pH value of the solution plays a significant role in the adsorption capacity of the adsorbate onto the adsorbent. As can be seen in Figure 5, with an increase in the initial pH of the MB solution from 3 to 8, adsorption capacity and efficiency increases rapidly and then increases slowly with a further increase in the pH. The maximum adsorption capacity was obtained at 1.245 g.g^−1^, with 99.64% efficiency at pH 11. This result can be explained by the electrostatic interaction between the cationic MB species and the surface of the adsorbent, which should be a negatively charged species. The lower adsorption at acidic pH levels was probably due to the presence of an excess of H^+^ ions competing with the dye cations for adsorption sites [19]. In order to confirm these results, the pHzpc of the onion membrane was determined. In this study, pHzpc value was 5.9, which at this pH the adsorbent surface has net electrical neutrality. At a pH below the pHzpc, the surface of the adsorbent is positive, and at a pH above the pHzpc, the surface of the adsorbent becomes more negatively charged by losing protons. Therefore, the adsorption of the MB reached its maximum value in the higher pH because of strong electrostatic attractions between the negatively charged surface of the onion membrane and the cationic MB.

#### Effect of adsorbent amount on adsorption

**Figure 6:**
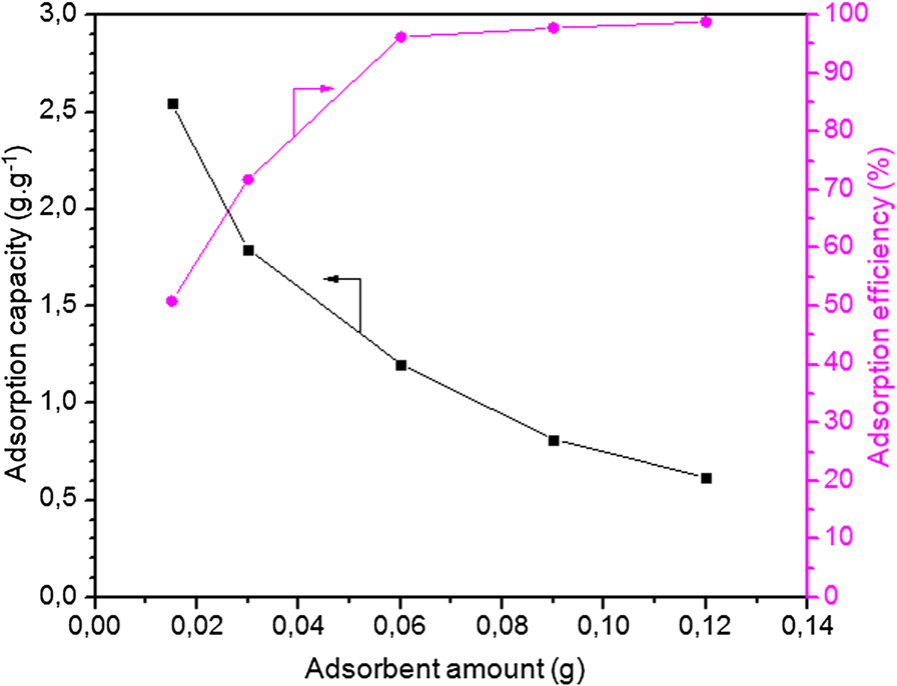
**Effect of adsorbent amount on the MB adsorption capacity (g.g** ^**−1**^ **) and efficiency (%) of membrane was plotted.** In these experiments, the membrane adsorbed dye molecules from a 0.3 g.L^−1^ dye solution (250 ml) with a pH of 7.1 at 20°C for 8 hours.

In order to find the influence of various onion membrane amounts on their dye uptake capacity, an adsorption of 250 mL MB solutions (0.3 g.L^−1^) was examined using five different membrane doses ranging from 0.015 to 0.12 g for eight hours in the atmospheric conditions. As shown in Figure 6, with an increase in the amount of membrane to 0.12 g, the adsorption amount and consequently the adsorption efficiency increases to 98.83%. This is most likely due to an increase in the numbers of adsorption sites at the adsorbent surface area, and as a result, increases the removal efficiency of MB. However, it is reasonable to observe a decrease in adsorption capacity to 0.617 g.g^−1^ by increasing the adsorbent dose, which is a denominator of the fraction in Equation 3.

#### Effect of the initial concentration of dye solution on adsorption

**Figure 7:**
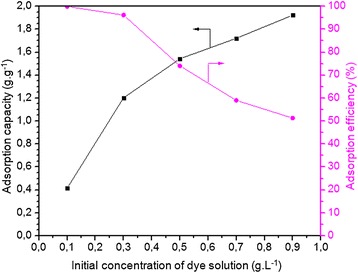
**Effect of initial dyes concentration on the MB adsorption capacity (g.g** ^**−1**^ **) and efficiency (%) of membrane was plotted.** In these experiments, 0.06 g of the membrane adsorbed dye molecules from a dye solution (250 ml) with a pH of 7.1 at 20°C for 8 hours.

**Figure 8:**
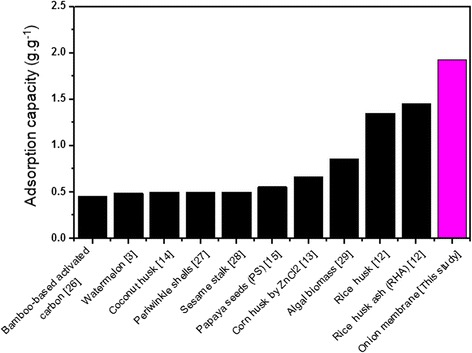
**The maximum MB adsorption capacities of onion membrane were compared with several biomass adsorbents.**

Figure 7 shows the maximum capacity of an onion membrane in the adsorption of MB using adsorbent-adsorbate solutions with different initial dye concentrations (0.1-0.9 g.L^−1^). It is clear that the MB adsorption capacity of an onion membrane increases with an increase in the initial concentration of the solution. This is most likely due to a greater availability of adsorbate ions in the vicinity of the onion membrane and a high driving force for mass transfer before the adsorption-desorption equilibrium was reached [25]. However, in this study, unlike the adsorption amount and capacity, the efficiency of the dye removal decreased, which may be attributed to the saturation of adsorption sites on the adsorbent surface [9]. The maximum MB adsorption capacity of an onion membrane is 1.9230 g.g^−1^ with the lowest efficiency at 51.28%, which is significantly higher than the other adsorbents [26-29] listed in Figure 8.

#### Isotherm of adsorption

**Figure 9:**
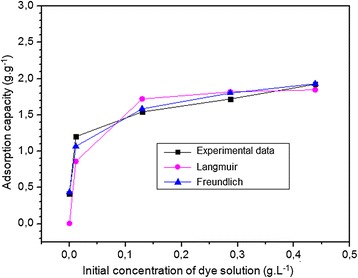
**Adsorption isotherm of MB on onion membrane by Langmuir and Freundlich were plotted.** In these experiments, 0.06 g of the membrane adsorbed dye molecules from a dye solution (250 ml) with a pH of 7.1 at 20°C for 8 hours.

The adsorption isotherm generally illustrates the interaction of an adsorbate with the adsorbent and also it can indicates the adsorption capacity of the adsorbent. Therefore, two isotherm models, Langmuir and Freundlich were investigated to find a more suitable model for the design process. The Langmuir model, the most popular, has been widely used to describe single-solute systems. The Langmuir model is based on the assumption that adsorbates produce monolayer coverage on the outer surface with uniform energies of adsorption, which is structurally homogeneous [30]. In the Freundlich model, the adsorption of an adsorbate occurs on a heterogeneous surface via multilayer adsorption with non-uniform distribution of heat of adsorption. The theoretical Langmuir and Freundlich isotherm models are represented by the following equations:

8 $$ \frac{{\mathrm{C}}_{\mathrm{e}}}{{\mathrm{q}}_{\mathrm{e}}}=\frac{{\mathrm{C}}_{\mathrm{e}}}{{\mathrm{q}}_{\mathrm{m}}}+\frac{1}{{\mathrm{q}}_{\mathrm{m}}{\mathrm{k}}_{\mathrm{L}}} $$

9 $$ {\mathrm{logq}}_{\mathrm{e}}={\mathrm{logk}}_{\mathrm{F}}+\frac{1}{\mathrm{n}}{\mathrm{logC}}_{\mathrm{e}} $$

where q_e_ (g.g^−1^) is the amount of dye adsorbed at equilibrium time, q_m_ (g.g^−1^) is the maximum adsorption capacity, and C_e_ (g.L^−1^) is the equilibrium dye concentration. k_L_ and k_F_ (L.g^−1^) are the Langmuir and Freundlich adsorption equilibrium constant. 1/n is the empirical Freundlich constant. As it is clear from Figure 9, the calculated values of q_e_ belong to the Freundlich model is in agreement with the experimental value, which revealed that the Freundlich model is more suitable than the Langmuir model for describing the adsorption. This is confirmed by the correlation coefficient (R^2^) of the Freundlich isotherm model (0.9922), which is greater than R^2^ (0.9878) of the Langmuir model (Table 2). The empirical Freundlich constant (1/n) which can be obtained from the linear plot of logq_e_ versus logC_e_, is an indicator of the favorability and surface affinity for the solute. When the 1/n values are in the range 0.1-1, the adsorption process is favorable. In addition, if the n is below one, then the adsorption is a chemical process; otherwise, the adsorption is a physical process. In this study, the value of 1/n is 0.24, lying between 0.1 and 1, indicating that adsorption of MB ions by an onion membrane is favorable with physisorption.

**Table 2:** **Comparison of isotherm models for adsorption of MB using onion membrane**

**Isotherm models and parameters**	**MB**
**Langmuir**	
q_max_ (g.g^−1^)	1.9054
q_e_ cal. (g.g^−1^)	1.8471
K_L_ (L.g^−1^)	72.387
R^2^	0.9878
**Fruendlich**	
q_e_ cal. (g.g^−1^)	1.9312
K_F_ (L.g^−1^)	2.2074
n	6.1690
R^2^	0.9922

#### Effect of temperature on adsorption

**Figure 10:**
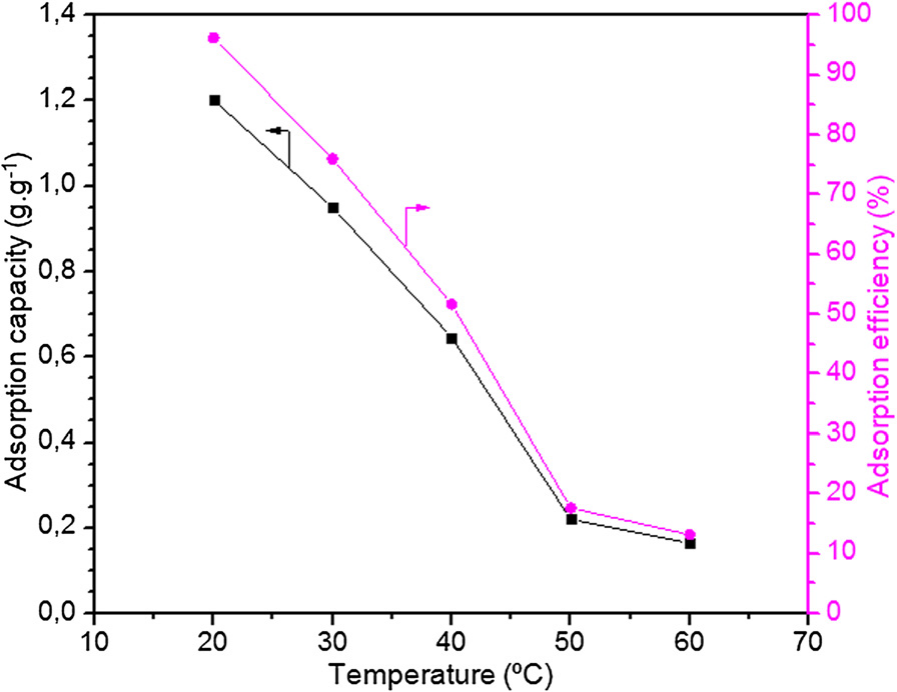
**Effect of temperature on the MB adsorption capacity (g.g** ^**−1**^ **) and efficiency (%) of membrane was plotted.** In these experiments, 0.06 g of membrane adsorbed dye molecules from a 0.3 g.L^−1^ dye solution (250 ml) with a pH of 7.1 for 8 hours.

The effect of temperature on the adsorption capacity of onion membrane (0.06 g) was studied using 250 mL of MB solution in different temperatures (20°C-60°C) for eight hours (480 min) in the atmospheric conditions. As shown in Figure 10, increasing the temperature leads to a decrease in the MB adsorption capacity of the membrane (0.164 g.g^−1^, with 13.15% efficiency) after eight hours of contact time. This can be attributed to a weakening of the adsorptive forces between the active sites on the sorbent and the dye molecules due to the degradation of the onion membrane in the high temperature. The results suggest that a high temperature is not suitable for adsorbing MB when the onion membrane is adsorbent. Therefore, it is better to let the temperature of industrial outcome solutions decrease to 20°C to achieve maximum adsorption capacity.

#### Adsorption thermodynamics

The effect of temperature can be further investigated by calculating the thermodynamic parameters, including standard enthalpy change (ΔH°), the Gibbs free energy change (ΔG°), and standard entropy change (ΔS°). They have been determined using the following equations:

10 $$ \varDelta {\mathrm{G}}^0=-\mathrm{R}\mathrm{T}\ { \ln \mathrm{K}}_{\mathrm{d}} $$

The distribution ratio (K_d_), can be calculated using the below equation:

11 $$ {\mathrm{K}}_{\mathrm{d}}=\frac{{\mathrm{q}}_{\mathrm{e}}}{{\mathrm{C}}_{\mathrm{e}}} $$

Then, the relation between ΔG°, ΔH°, and ΔS° can be expressed by the following equations:

12 $$ \varDelta \mathrm{G}{}^{\circ}=\varDelta \mathrm{H}{}^{\circ}-\mathrm{T}\varDelta \mathrm{S}{}^{\circ} $$

Standard enthalpy (ΔH°) and entropy (ΔS°) were determined from the slope and intercept of the plot of lnK_d_ vs 1/T, which came from the Van’s Hoff equation:

13 $$ { \ln \mathrm{K}}_{\mathrm{d}}=\frac{\varDelta {\mathrm{S}}^{{}^{\circ}}}{\mathrm{R}}-\frac{\varDelta {\mathrm{H}}^{{}^{\circ}}}{\mathrm{R}\mathrm{T}} $$

Table 3 presents the thermodynamic parameters of MB adsorption by the onion membrane in different contact temperatures. It is clear that the values of free energy (ΔG°) at all temperatures except 60°C were negative. This confirms that the adsorption of dye at these temperatures is spontaneous and thermodynamically favorable. The negative value of ΔH° confirms the exothermic nature of adsorption, which is supported by the decrease in the adsorption capacity of the onion membrane. Besides, the negative value of ΔS° resulted from the decreased randomness at the solid–liquid interface during the adsorption of MB ions onto the onion membrane.

**Table 3:** **Thermodynamic parameters for adsorption of MB using onion membrane**

**Thermodynamic constant**	**Temperature (K)**				
	**293**	**303**	**313**	**323**	**333**
ΔG° (kJ.mol^−1^)	−10.79	−7.533	−4.273	−1.013	2.247
ΔH° (kJ.mol^−1^)	−106.3				
ΔS° (kJ.mol^−1^.K^−1^)	−0.326				

## Conclusion

The present study confirms the high potential of onion membranes with special physical and chemical characteristics for quick and efficient removal of MB from aqueous solutions. The amount of dye adsorbed varied with time, temperature, pH, adsorbent dosage, and initial dye concentration. The adsorption experiments indicated that onion membrane have a high MB adsorption capacity (1.9230 g.g^−1^) when 0.06 g of adsorbent was immersed in 250 ml of dye solution (0.9 g.L^−1^) with a pH of 7.1 at 20°C. The adsorption of MB by the onion membrane agreed with the pseudo-second-order model. Moreover, analysis of the equilibrium isotherms using the Langmuir and Freundlich isotherms showed that the Freundlich model fitted well with the experimental data. The thermodynamic studies suggested that the adsorption reaction was an exothermic and spontaneous process. Finally, the results suggest the synthesis of polymer-based membranes with similar physical and chemical properties of onion membrane as valuable and highly efficient adsorbents, which can be applied for dye removal in water treatment processes.

## References

[1] Bayramoglu G, Adiguzel N, Ersoy G, Yilmaz M, Arica MY (2013). Removal of textile dyes from aqueous solution using amine-modified plant biomass of a. caricum: equilibrium and kinetic studies. Water Air Soil Pollut.

[2] Crini G (2005). Recent developments in polysaccharide-based materials used as adsorbents in waste water treatment. Prog Polym Sci.

[3] Lakshmipathy R, Sarada NC (2013). Adsorptive removal of basic cationic dyes from aqueous solution by chemically protonated watermelon (Citrullus Lanatus) rind biomass. Desalin Water Treat.

[4] Santhi T, Manonmani S (2012). Adsorption of methylene blue from aqueous solution onto a waste aquacultural shell powders (Prawn Waste). Sustain Environ Res.

[5] Constantin M, Asmarandei I, Harabagiu V, Ghimici L, Ascenzi P, Fundueanu G (2013). Removal of anionic dyes from aqueous solutions by an ion-exchanger based on pullulan microspheres. Carbohydr Polym.

[6] Saber-Samandari S, Saber-Samandari S, Nezafati N, Yahya K (2014). Efficient removal of lead (II) ions and methylene blue from aqueous solution using Chitosan/Fe-Hydroxyapatite nanocomposite beads. J Environ Manage.

[7] Mishra G, Tripathy M (1993). A critical review of the treatment for decolorization of textile effluent. Colourage.

[8] Eren E (2009). Investigation of a basic dye removal from aqueous solution onto chemically modified Unye Bentonite. J Hazard Mater.

[9] Salleh MAM, Mahmoud DK, Karim WAWA, Idris A (2011). Cationic and anionic dye adsorption by agricultural solid wastes: a comprehensive review. Desalination.

[10] Saber-Samandari S, Gulcan HO, Saber-Samandari S, Gazi M (2014). Efficient removal of anionic and cationic dyes from an aqueous solution using pullulan-graft-polyacrylamide porous hydrogel. Water Air Soil Pollut.

[11] Vadivelan V, Kumar KV (2005). Equilibrium, kinetics, mechanism, and process design for the sorption of methylene blue onto rice husk. J Colloid Interface Sci.

[12] Sharma P, Kaur R, Baskar C, Chung WJ (2010). Removal of methylene blue from aqueous waste using rice husk and rice husk ash. Desalination.

[13] Khodaie M, Ghasemi N, Moradi B, Rahimi M. Removal of Methylene Blue from Wastewater by Adsorption onto ZnCl_2_ Activated Corn Husk Carbon Equilibrium Studies. J Chemistr. 2013;383985.

[14] Khodaie M, Ghasemi N, Moradi B, Rahimi M. Removal of Methylene Blue from Wastewater by Adsorption onto ZnCl_2_ Activated Corn Husk Carbon Equilibrium Studies. J Chemistr. 2013; Article ID 383985, 6 pages, http://dx.doi.org/10.1155/2013/383985.

[15] Hameed BH (2009). Evaluation of papaya seeds as a novel nonconventional low-cost adsorbent for removal of methylene blue. J Hazard Mater.

[16] Saber-Samandari S, Gazi M, Yilmaz O (2013). Synthesis and characterization of chitosan-graft-poly (N-Allyl Maleamic Acid) hydrogel membrane. Water Air Soil Pollut.

[17] Sharma K, Kaith BS, Kumar V, Kalia S, Kumar V, Swart HC (2014). Water retention and dye adsorption behavior of Gg-Cl-Poly(Acrylic Acid-Aniline) based conductive hydrogels. Geoderma.

[18] Sartape AS, Patil SA, Patil SK, Salunkhe ST, Kolekar SS (2013). Mahogany fruit shell: a new low-cost adsorbent for removal of methylene blue dye from aqueous solutions. Desalin Water Treat.

[19] Saka C, Sahin O, Celik MS (2012). The removal of methylene blue from aqueous solutions by using microwave heating and pre-boiling treated onion skins as a new adsorbent. Energ Source Part A.

[20] Saka C, Sahin O (2011). Removal of methylene blue from aqueous solutions by using cold plasma- and formaldehyde-treated onion skins. Color Technol.

[21] Chowdhury A, Bhowal A, Datta S (2012). Equilibrium, thermodynamic and kinetic studies for removal of copper (II) from aqueous solution by onion and garlic skin. Water.

[22] Pathani D, Sharma S, Singh P. Removal of Methylene Blue by Adsorption onto Activated Carbon Developed from Ficus Carica Bast. Arab J Chem. 2013; Received 9 May 2012; accepted 17 April 2013. doi:10.1016/j.arabjc.2013.04.021.

[23] Suleria HAR, Butt MS, Anjum FM, Saeed F, Khalid N (2015). Onion: nature protection against physiological threats. Crit Rev Food Sci.

[24] Al-Ghouti MA, Khraisheh MAM, Ahmad MNM, Allen S (2009). Adsorption behaviour of methylene blue onto Jordanian diatomite: a kinetic study. J Hazard Mater.

[25] Saber-Samandari S, Saber-Samandari S, Gazi M (2013). Cellulose-Graft-Polyacrylamide/Hydroxyapatite composite hydrogel with possible application in removal of Cu(II) ions. React Funct Polym.

[26] Hameed BH, Din ATM, Ahmad AL (2007). Adsorption of methylene blue onto bamboo-based activated carbon: kinetics and equilibrium studies. J Hazard Mater.

[27] Bello OS, Adeogun IA, Ajaelu JC, Fehintola EO (2008). Adsorption of methylene blue onto activated carbon derived from periwinkle shells: kinetics and equilibrium studies. Chem Ecol.

[28] Maiti S, Purakayastha S, Ghosh B (2008). Production of low-cost carbon adsorbents from agricultural wastes and their impact on dye adsorption. Chem Eng Comm.

[29] Rubin E, Rodriguez P, Herrero R, Vicente MES (2010). Adsorption of methylene blue on chemically modified algal biomass: equilibrium, dynamic and surface data. J Chem Eng Data.

[30] Mahmoodi NM, Arami M, Bahrami H, Khorramfar S (2011). The effect of pH on the removal of anionic dyes from colored textile wastewater using a biosorbent. J Appl Polym Sci.

